# ‘Bit’ off more than he could chew: asymptomatic foreign body in the appendix

**DOI:** 10.1093/jscr/rjae109

**Published:** 2024-03-07

**Authors:** Zachary M S Waarala, Christopher Santucci, Jay H Sandberg

**Affiliations:** Michigan State University College of Osteopathic Medicine, 965 Wilson Rd, East Lansing, MI 48824, United States; Nova Southeastern University, Dr. Kiran C. Patel College of Osteopathic Medicine, 3200 S University Dr, Fort Lauderdale, FL 33328, United States; Michigan State University College of Osteopathic Medicine, 965 Wilson Rd, East Lansing, MI 48824, United States; Oakland Medical Center, 115 E Long Lake Rd, Troy, MI 48085, United States

**Keywords:** drill bit, appendix, appendicitis, foreign body

## Abstract

This report details the case of a 69-year-old male who had presented to the emergency department at the suggestion of his dentist after ingesting a diamond-tipped drill bit during a routine dental procedure. Through the use of radiograph, computed tomography, and colonoscopy, the drill bit was determined to be lodged in the distal vermiform appendix. Throughout his clinical course, the patient remained asymptomatic but was monitored closely for signs of complications of a retained foreign body in the appendix. Gastroenterology and general surgery were consulted on the case but ultimately non-surgical approaches prevailed and the drill bit passed in the stool. This patient’s case highlights the success of noninvasive measures for appendiceal foreign body removal.

## Introduction

The appendix is a small physiologic outpouching of the posteromedial cecum. While rare, ingested foreign bodies can become lodged in the appendix and predispose the patient to appendicitis. Several cases have been documented of an ingested dental drill bit becoming lodged in the lumen of the appendix [1–3]. Importantly, a review of appendiceal foreign bodies has shown that sharp, thin, stiff, and pointed objects increase the likelihood for life-threatening complications of the retained foreign body [4]. If the ingestion is known, patients should seek medical treatment for observation and potential prophylactic appendectomy. The foreign body should be monitored with serial abdominal radiographs and the patient regularly evaluated for signs of progression to appendicitis or complications thereof which should be managed surgically. The current guideline is to manage these patients with appendectomy even if asymptomatic [5]. This case report documents the success of non-surgical management in one instance of a swallowed dental drill bit that became lodged in the appendix.

## Case presentation

The patient is a 69-year-old male with past medical history significant for bicuspid aortic valve, essential hypertension on chlorthalidone 25 mg daily, hyperlipidemia on pravastatin 40 mg daily, and hypothyroidism on levothyroxine 125 mcg daily. The patient has not had any surgeries. Social history reveals occasional alcohol use and the patient is a never-smoker. The patient’s most recent colorectal cancer screening with Cologuard 6 months before presentation was negative. The most recent colonoscopy, completed 5 years before presentation, was negative.

The patient swallowed a diamond-tipped drill bit while undergoing routine dental work. The patient’s dentist suggested that the patient be evaluated in the emergency department if the drill bit had not passed within 24 hours and instructed the patient to take laxatives and search stool content for the drill bit. The patient presented the next day to the emergency department for evaluation as the drill bit had not yet passed despite Dulcolax use. At this time, he remained asymptomatic. In the emergency department, vital signs were obtained and revealed blood pressure 164/88 mmHg, pulse rate of 82 beats per minute, respiratory rate of 20 breaths per minute, oxygen saturation 97% on room air, and oral temperature of 36.8°C (98.3 degrees Fahrenheit). On physical exam, the patient was nontoxic and stable. The abdomen was soft, non-distended, and nontender to palpation without rebound or guarding. The lungs were clear to auscultation bilaterally and the heart was of regular rate and rhythm.

**Figure 1:**
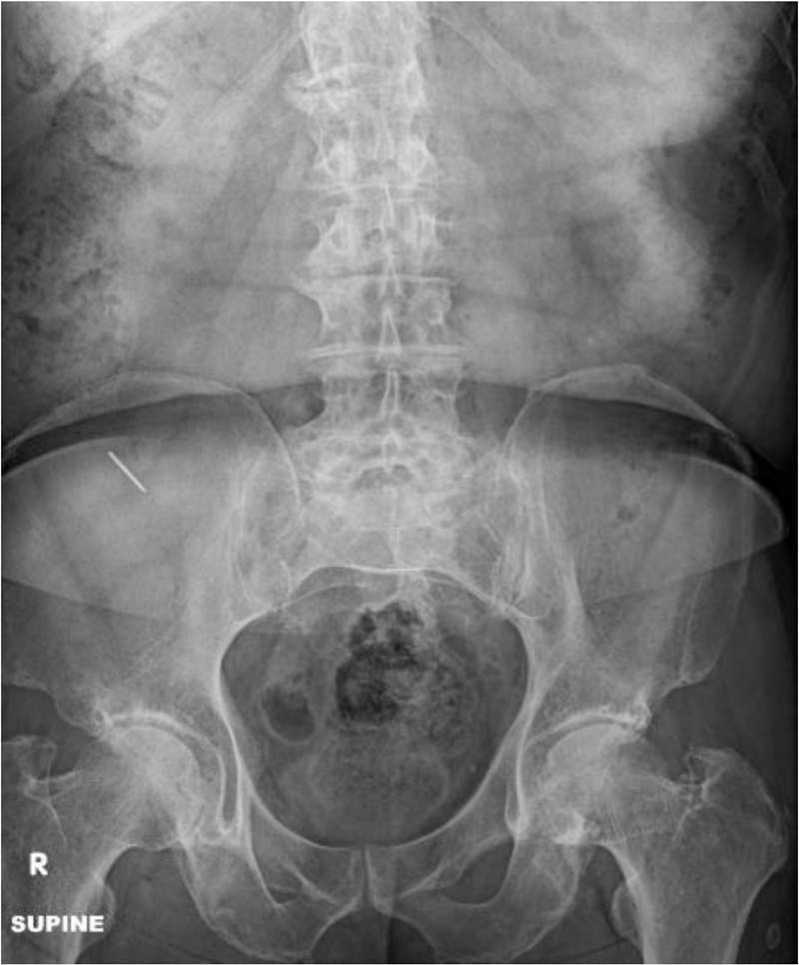
Abdominal radiograph shows a 2.3 cm linear radiopaque density in the right lower quadrant.

A radiograph of the abdomen was performed which revealed a 2.3 cm linear radiopaque density at the right lower quadrant overlying the right iliac wing (Fig. 1). The gastrointestinal service was consulted and the patient was admitted to the hospital for observation and to undergo colonoscopy the following morning. The patient was started on bowel preparations and otherwise given nothing by mouth.

The patient had no acute events overnight and remained asymptomatic. Perianal and digital rectal exams were normal. The colonoscopy was performed and revealed several diverticuli but no foreign body was seen in either the colon or the distal 30 cm of the small intestine. An abdominal radiograph was performed after the colonoscopy and again showed a 2.3 cm linear radiopaque density in the right lower quadrant consistent with ingested metallic foreign body. There was no change in appearance or location of the foreign body as compared with the prior radiograph.

The patient remained asymptomatic. Regular exam of the stool in the hospital was negative for blood or passage of the drill bit. The patient remained hemodynamically stable throughout the hospital stay and laboratory studies including a complete blood count and comprehensive metabolic panel were within normal limits. Surgery was consulted, who recommended outpatient evaluation. The patient was discharged home on polyethylene glycol 3350 17 grams once per day by mouth and instructed to follow with surgery outpatient to repeat abdominal radiograph.

**Figure 2:**
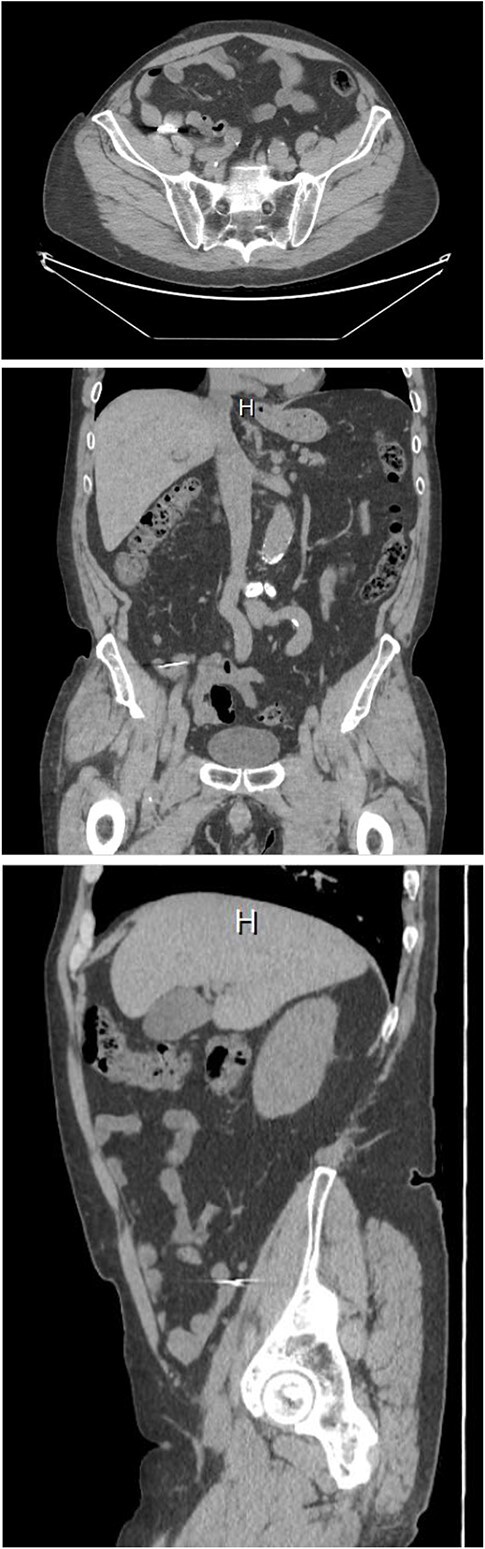
Computed tomography of the abdomen, axial, coronal, and sagittal views. The foreign body lies over the distal appendix. Detail is limited due to artifact.

Two weeks after discharge, the patient met with the surgical service and underwent computed tomography (CT) imaging (Fig. 2). Given the negative colonoscopy and these findings on CT, the foreign body was posited to be within the lumen of the appendix. Still, the patient was asymptomatic and regular stool examination was negative for blood or foreign body material. The surgical service recommended that the patient undergo removal of the appendix in accordance with current guidelines to avoid perforation. The appendectomy was scheduled and the patient continued serial abdominal radiographs and bowel regimen in the interim. The preoperative abdominal radiograph taken before the scheduled appendectomy showed absence of the foreign body. The patient admits to recent lapse in examining his stool for presence of the foreign body, and it was assumed that the patient had passed the drill bit.

## Discussion

This case report documents an instance of non-surgical management of a patient with an ingested dental drill bit that became lodged in the distal lumen of the appendix. Because of the propensity for perforation and other complications of acute appendicitis secondary to ingested foreign body, most cases of an ingested drill bit in the appendix are managed surgically. However, in a patient without radiographic or clinical signs of appendicitis, this case report shows that management with laxatives, serial abdominal radiographs, and patient education may be a viable alternative.
